# Supplementary figures and images for: The Cell Ontology in the age of single-cell omics
Source: Sci Data. 2026 Apr 24;13:946. doi: 10.1038/s41597-026-07173-8 (PMC13315338; doi:10.1038/s41597-026-07173-8)

A

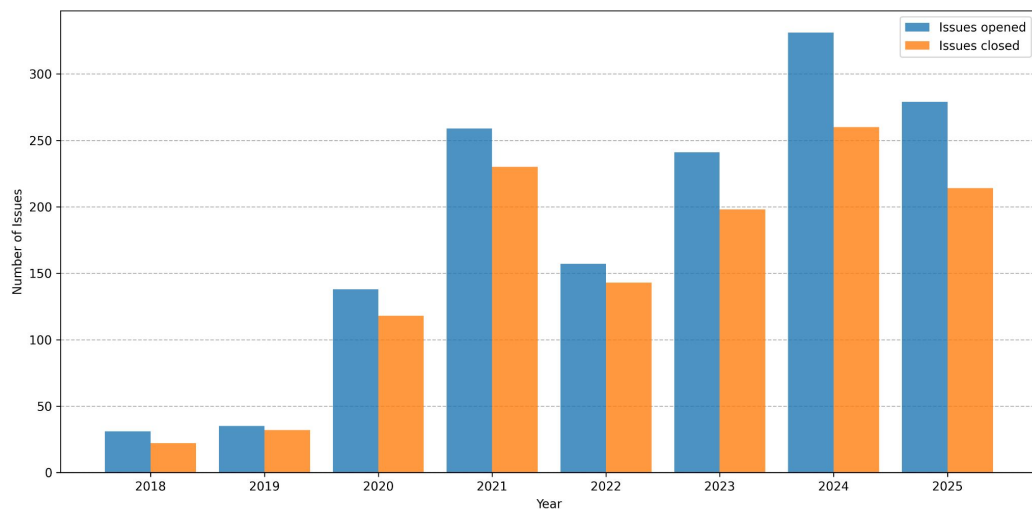

B

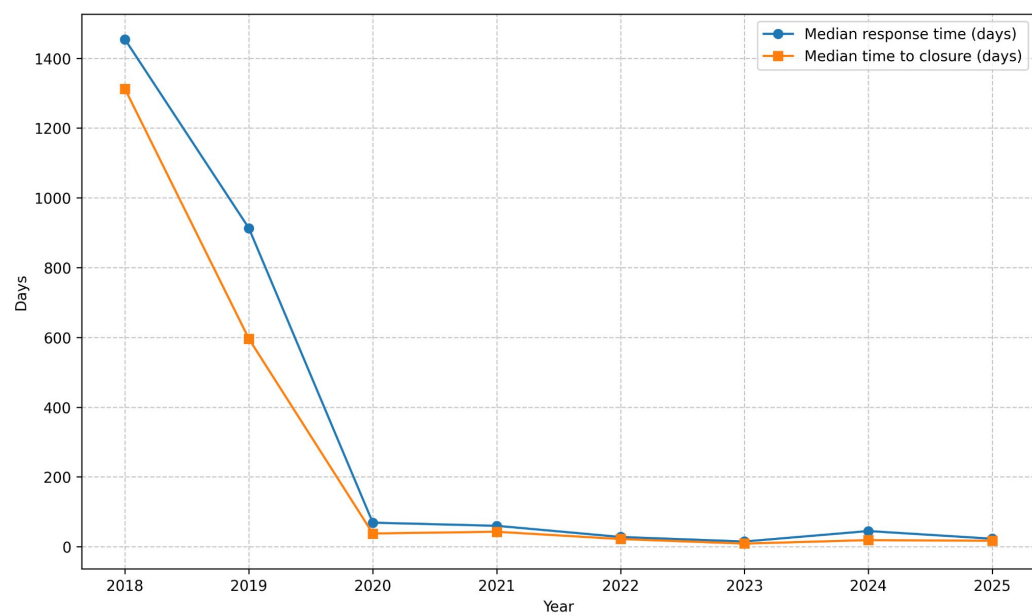

C

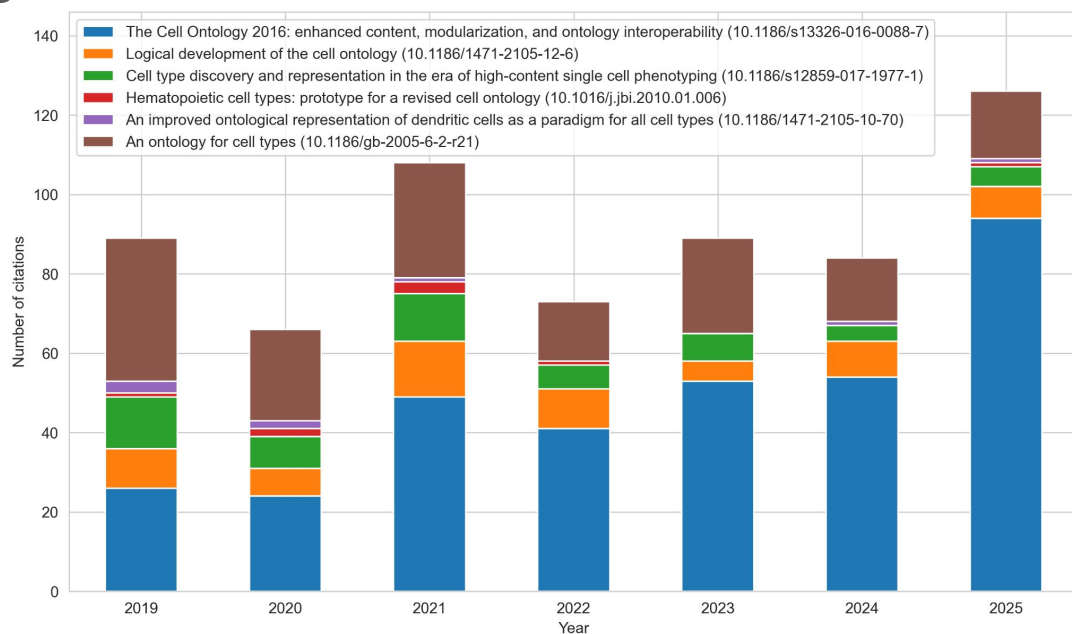

Supplement: Supplementary file 1 — Supplementary Figure S1 [file 41597_2026_7173_MOESM1_ESM.pdf]
