# Supplementary material for: The Cell Ontology in the age of single-cell omics
Source: Sci Data. 2026 Apr 24;13:946. doi: 10.1038/s41597-026-07173-8 (PMC13315338; doi:10.1038/s41597-026-07173-8)
Supplement: Supplementary file 2 — Supplemental Table S1 [file 41597_2026_7173_MOESM2_ESM.doc]

| Project | Description | CL use | Link/Citation |
| --- | --- | --- | --- |
| HuBMAP, Human Reference Atlas | A consortium of research teams that aims to develop a framework for mapping the human body at single-cell resolution. | Maps all its cell types to CL and PCL. | [1] |
| Human Cell Atlas (HCA), Data Coordination Platform | A consortium of researchers that aim to create a comprehensive reference map of every cell type in the human body. | Uses CL to annotate data from cell suspension studies. | [2] |
| CZ CELLxGENE | A Web platform (CZ CELLxGENE Discover) that hosts a massive collection of single cell RNAseq data. | Uses CL to annotate cell type and CL structure to drive Findability and Interoperability. | [3] |
| BGEE | A web platform integrating curated gene expression data across multiple modalities and species. | Uses CL to annotate datasets. | [4] |
| Cell Annotation Platform | A platform for community annotation of scRNAseq datasets. | Uses an autosuggest system to recommend CL terms for annotation leveraging relationships with Uberon tissue terms. | [https://celltype.info](https://celltype.info/) |
| Single Cell Expression Atlas | An online bioinformatics resource that enables easy access to information about gene expression across species, tissues, cells, experimental conditions and diseases. | Uses CL and its extensions to annotate scRNAseq data and "anatamogram" schematics illustrating cell type location in organs and tissues. | [5] |
| Brain Data Standards Ontology | A data-driven ontology, with an initial focus on the data generated in the BRAIN Initiative Cell Census Network (BICCN) mini-atlas of the mammalian primary motor cortex. | Uses CL terms as superclasses. | [6] |
| CellTypist | A machine learning driven annotation transfer system for annotating cell types in single cell transcriptomic datasets. | All cell types defined by the CellTypist model are linked to CL terms. | [7] |
| HuBMAP, Azimuth | An online platform that annotates uploaded datasets using annotation transfer from reference datasets. | Uses CL to annotate reference datasets. | https://azimuth.hubmapconsortium.org/ |
| Ontology-based single cell Classification (OnClass) | An algorithm and accompanying software for automatically classifying cells from scRNAseq experiments. | Uses CL graph to infer cell type relationships. | [8] |
| Sfaira | A single-cell data zoo for public data sets paired with a model zoo for executable pre-trained models. | Uses CL to annotate cell type terms and the ontology graph to train a supervised model for cell type identification. | [9] |
| Cell BLAST | A cell-querying method built on a neural network-based generative model and a customized cell-to-cell similarity metric that aims to be a one-stop solution for real-world scRNA-seq cell querying and annotation. | Uses CL to annotate cell types and ontology structure for improved assignment. | [10] |
| CellMarker 2.0 | A database of Cell Markers. | Uses CL and Uberon to record cell type markers and their tissue context in human and mouse. Data is curated from the literature and supplemented by integrated datasets and data analysis tools. | [11] |
| Cell Image Library | An online resource of images of cells, their substructures and phenotypes. | Uses CL in annotation of microscopy images. | [12] |
| human Ensemble Cell Atlas (hECA) | A unified informatics framework for seamless cell-centric data assembly. | Maps names to CL terms. | [13] |
| celldex | An R package containing reference datasets with cell type information (“Pokédex for Cell Types”). | Maps names to CL terms. | https://bioconductor.org/packages/devel/data/experiment/vignettes/celldex/inst/doc/userguide.html |
| Cellarium | A machine learning driven annotation transfer system that leverages the CELLxGENE scRNAseq corpus and its ontology annotation. | Leverages CL annotation on CELLxGENE. Outputs annotation with CL terms. | <https://cellarium.ai/tool/cellarium-cell-annotation-service-cas/> |
| popV | Annotation transfer with Confidence scores. Polls the results of multiple annotation transfer algorithms including OnClass. | Uses CL as both input and output. | [14] |

**Table 2.** A non-exhaustive list of resources that use the Cell Ontology.

# Supplemental References

1. [HuBMAP Consortium. The human body at cellular resolution: the NIH Human Biomolecular Atlas Program. *Nature* **574**, 187–192 (2019).](http://paperpile.com/b/JEb01J/myNa)

2. [Regev, A. *et al.* The Human Cell Atlas. *Elife* **6**, (2017).](http://paperpile.com/b/JEb01J/OfN5I)

3. [CZI Single-Cell Biology Program *et al.* CZ CELL×GENE Discover: A single-cell data platform for scalable exploration, analysis and modeling of aggregated data. *bioRxiv* 2023.10.30.563174 (2023) doi:](http://paperpile.com/b/JEb01J/swO9i)[10.1101/2023.10.30.563174](http://dx.doi.org/10.1101/2023.10.30.563174)[.](http://paperpile.com/b/JEb01J/swO9i)

4. [Bastian, F. B. *et al.* The Bgee suite: integrated curated expression atlas and comparative transcriptomics in animals. *Nucleic Acids Res* **49**, D831–D847 (2021).](http://paperpile.com/b/JEb01J/sND3)

5. [George, N. *et al.* Expression Atlas update: insights from sequencing data at both bulk and single cell level. *Nucleic Acids Res.* **52**, D107–D114 (2024).](http://paperpile.com/b/JEb01J/qLXqU)

6. [Tan, S. Z. K. *et al.* Brain Data Standards - A method for building data-driven cell-type ontologies. *Sci. Data* **10**, (2023).](http://paperpile.com/b/JEb01J/r6zYF)

7. [Domínguez Conde, C. *et al.* Cross-tissue immune cell analysis reveals tissue-specific features in humans. *Science* **376**, eabl5197 (2022).](http://paperpile.com/b/JEb01J/Wwj3i)

8. [Wang, S. *et al.* Leveraging the Cell Ontology to classify unseen cell types. *Nat. Commun.* **12**, 5556 (2021).](http://paperpile.com/b/JEb01J/H66da)

9. [Fischer, D. S. *et al.* Sfaira accelerates data and model reuse in single cell genomics. *Genome Biol.* **22**, 248 (2021).](http://paperpile.com/b/JEb01J/yusnX)

10. [Cao, Z.-J., Wei, L., Lu, S., Yang, D.-C. & Gao, G. Searching large-scale scRNA-seq databases via unbiased cell embedding with Cell BLAST. *Nat. Commun.* **11**, 3458 (2020).](http://paperpile.com/b/JEb01J/vH38M)

11. [Hu, C. *et al.* CellMarker 2.0: an updated database of manually curated cell markers in human/mouse and web tools based on scRNA-seq data. *Nucleic Acids Res.* **51**, D870–D876 (2023).](http://paperpile.com/b/JEb01J/Yqh5d)

12. [Orloff, D. N., Iwasa, J. H., Martone, M. E., Ellisman, M. H. & Kane, C. M. The cell: an image library-CCDB: a curated repository of microscopy data. *Nucleic Acids Res.* **41**, D1241–50 (2013).](http://paperpile.com/b/JEb01J/3Ojud)

13. [Chen, S. *et al.* hECA: The cell-centric assembly of a cell atlas. *iScience* **25**, 104318 (2022).](http://paperpile.com/b/JEb01J/2fPYx)

14. [Ergen, C. *et al.* Consensus prediction of cell type labels in single-cell data with popV. *Nat. Genet.* (2024) doi:](http://paperpile.com/b/JEb01J/3SS4G)[10.1038/s41588-024-01993-3](http://dx.doi.org/10.1038/s41588-024-01993-3)[.](http://paperpile.com/b/JEb01J/3SS4G)
